# ﻿*Rhododendron mulunense* (Ericaceae), a new species from the karst mountains of Guangxi and Guizhou provinces, China

**DOI:** 10.3897/phytokeys.269.175011

**Published:** 2026-01-08

**Authors:** Ying Hou, Yu-Song Huang, Liu-Juan Luo, Zheng-Ren Chen, Xing-Xing Mao

**Affiliations:** 1 College of Life Sciences, Zhejiang Normal University, Jinhua 321004, Zhejiang, China; 2 Xingzhi College, Zhejiang Normal University, Lanxi 321100, Zhejiang, China; 3 Guangxi Key Laboratory of Functional Phytochemicals Research and Utilization, Guangxi Institute of Botany, Guangxi Zhuang Autonomous Region and the Chinese Academy of Sciences, Guilin, 541006, Guangxi, China; 4 University of Chinese Academy of Sciences, Beijing, 100049, China; 5 Administrative Centre of Guangxi Mulun National Nature Reserve, Huanjiang 547100, Guangxi, China; 6 Administrative Centre of Mao-lan National Nature Reserve, Libo 558400, Guizhou, China

**Keywords:** Conservation, morphology, *Rhododendron mulunense*, *Rhododendron wumingense*, taxonomy

## Abstract

*Rhododendron mulunense*, a new species of *Rhododendron* (Ericaceae) within subsect. Maddenia in sect. Rhododendron and endemic to karst areas of Guangxi and Guizhou Province, SW China, is described and illustrated. The new species most closely resembles *R. wumingense*, particularly in scale density on the abaxial leaf surface and in flower color and shape, but can be readily distinguished by its hairless shoots and leaves, larger tubular-funneled corolla, and longer and thinner white tube flushed with pink. According to the IUCN Red List Categories and Criteria, the threatened status of the new species is assessed as “Endangered, EN C2a(i), D.” We provide detailed documentation of its morphological characteristics, geographical distribution, digitized holotype voucher, and comparative photographs, along with a diagnostic table. An identification key is provided as a primary tool to enable taxonomic discrimination of this species from its congeners.

## ﻿Introduction

*Rhododendron* L. (1753: 392) is the largest genus in the family Ericaceae, comprising approximately 1,150 species worldwide (Khan et al. 2021) and over 1,000 species in the northern hemisphere (Feng 1983; Chamberlain et al. 1996; Gibbs et al. 2011; Geng 2014; Yu et al. 2017; Mao et al. 2021). The species were traditionally divided into eight subgenera (Chamberlain et al. 1996), of which only five are supported by phylogenetic studies (Goetsch et al. 2005; Ma et al. 2022; Xia et al. 2022, 2024). The great majority of *Rhododendron* species occur in China and the Malay Archipelago (Fang and Min 1995; Brown et al. 2006). In China, there are ca. 600 species of the genus documented in most provinces except for Ningxia and Xinjiang (Fang et al. 2005; Cheng et al. 2021), among which ≥ 400 species are endemic, with the greatest species diversity in the south and southwest of the country (Chen and Fang 1957; Yang et al. 1994; Li 1995; Fang et al. 2005; Cheng et al. 2021).

Field investigations in Guangxi and Guizhou Province, southern China, have led to the discovery of several new *Rhododendron* species in recent years (Chen and Lan 2003; Gao and Li 2004; Chen et al. 2010a, b; Ma et al. 2015; Dai et al. 2020; Tong et al. 2020; Chang et al. 2021; Deng et al. 2025), indicating that the species diversity of *Rhododendron* in these areas has been underestimated. During fieldwork investigating *Rhododendron* diversity in Guangxi Mulun National Nature Reserve from 2021 to 2024, we found an unknown *Rhododendron* species growing as an upright shrub on a karst mountain. It had scaly and broad leaves, terminal and umbellate inflorescences, large-sized and tubular-funnelform corollas with five lobes, 10 unequal stamens, scaly and slender styles, and large and scaly capsules – typical of subsection Maddenia (Hutch.) Sleumer (1949: 525) in subgenus Rhododendron. Then, in late March 2025, we obtained photographs of *Rhododendron* plants with matching morphological characteristics from the karst mountains of Napo County, southwest Guangxi, and from Maolan National Nature Reserve, southeast Guizhou. We compared the plants both to descriptions in the literature and to herbarium specimens of species in subsect. Maddenia (Fang et al. 2005; Li 2008; Geng 2014; Chang et al. 2021; Yang et al. 2022), concluding that they represent a species that is new to science. We formally describe and illustrate the species here.

## ﻿Materials and methods

Morphological studies of the newly identified species were conducted through field investigations during flowering and fruiting times at type localities, and through thorough examinations of herbarium specimens of related species in the herbaria of the Guangxi Institute of Botany (**IBK**), South China Botanical Garden Herbarium (**IBSC**), Kunming Institute of Botany (**KUN**), and Institute of Botany (**PE**), Chinese Academy of Sciences (Qin et al. 2019), as well as online images of specimens in the herbaria of The Natural History Museum (**BM**), Royal Botanic Garden Edinburgh (**E**), and Royal Botanic Gardens (K) (https://sweetgum.nybg.org/science/ih/), and on the website of JSTOR Global Plants (https://plants.jstor.org/). Voucher specimens of the new species are deposited in the Herbarium of the Guangxi Institute of Botany (IBK), Chinese Academy of Sciences (Qin et al. 2019). In addition to consulting relevant publications and herbarium specimens (including types) of the closely related species *Rhododendron wumingense* (Fang 1983) in the herbarium (IBK), we conducted field observations for *R. wumingense* in spring 2024. We then performed comparative analyses of morphological characteristics and phenological patterns for the newly described species and related species. The conservation status of the new species was assessed based on the IUCN Red List Categories and Criteria (Gibbs et al. 2011; IUCN 2012).

## ﻿Taxonomic treatment

### ﻿ Rhododendron mulunense

Taxon classificationPlantaeEricalesEricaceae

Y.S.Huang & X.X.Mao sp. nov.

13369960-870B-5345-83A5-6540EBDEEDD2

urn:lsid:ipni.org:names:77374716-1

**Figure 1.**
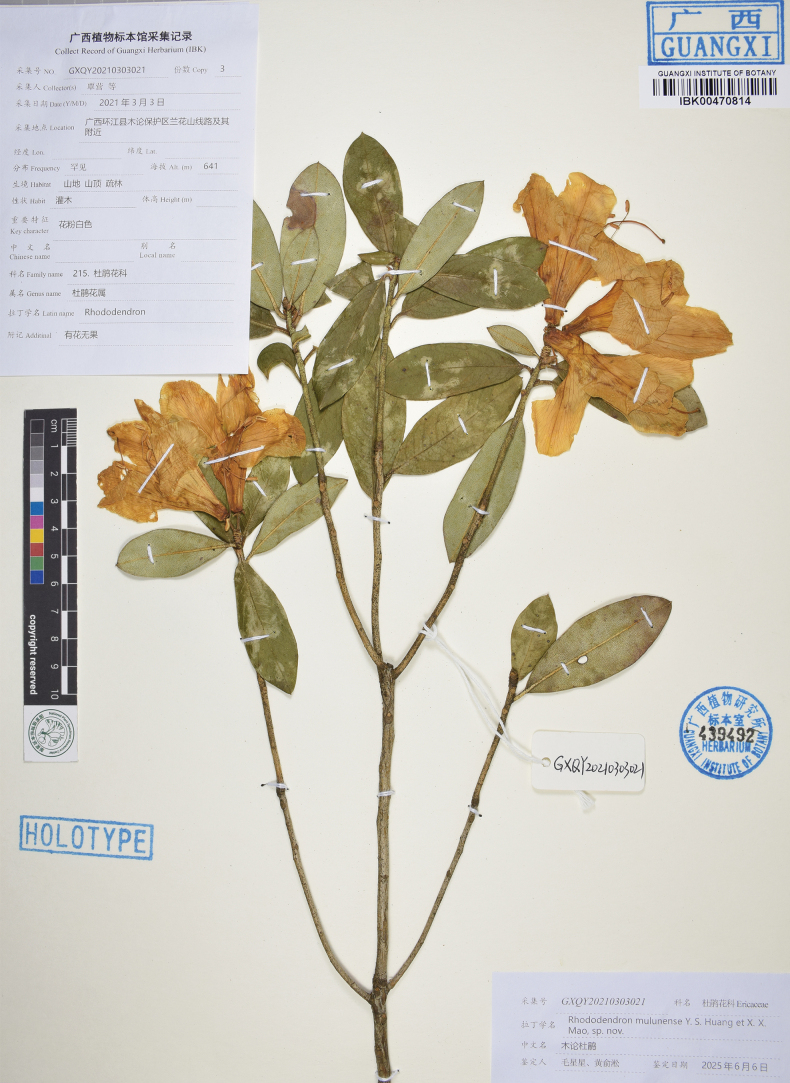
Holotype specimen of *Rhododendron mulunense* (IBK).

**Figure 2.**
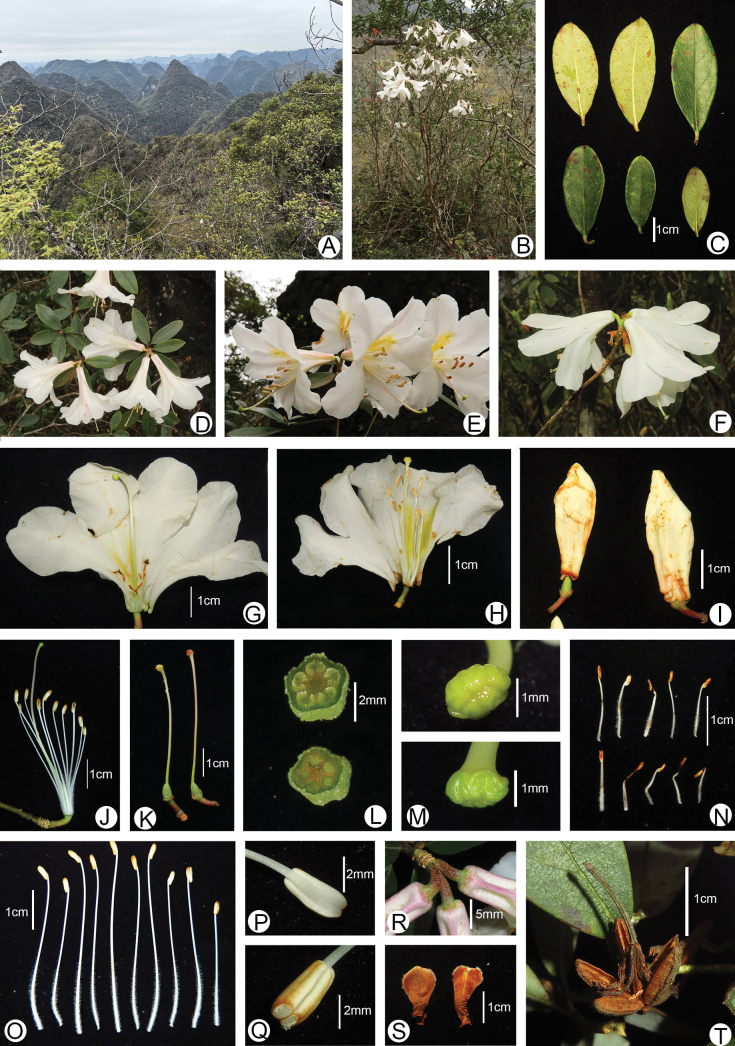
*Rhododendron mulunense*. **A.** The habitat of this new species in type localities; **B.** Flowering plants; **C.** Leaves; **D–F.** Flowering branchlets, inflorescences, and flowers; **G, H.** Dissection of corolla; **I.** Flower buds; **J.** Stamens and style; **K.** Styles and ovaries; **L.** Dissection of ovary; **M.** Stigmas; **N.** Poorly developed stamens; **O.** Normal stamens; **P, Q.** Anther abaxial and adaxial surface; **R.** Tube base; **S.** Flower bud scales; **T.** Dehiscent capsule. Photographs by Yu-Song Huang.

**Figure 3.**
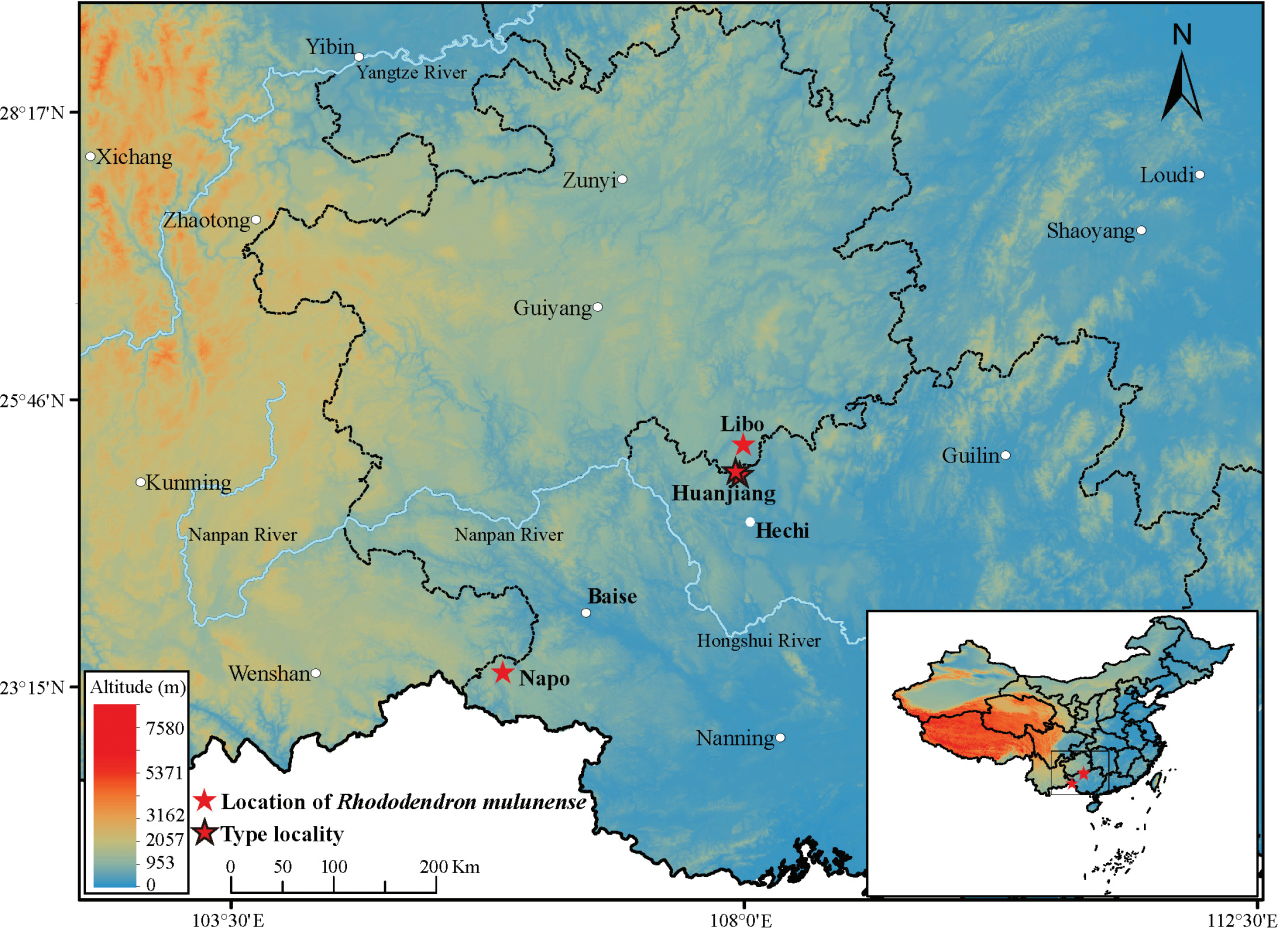
The known geographical distribution of *Rhododendron mulunense* in Guangxi and Guizhou Province, SW China (two localities at Libo in Guizhou are superimposed). Photograph by Xing-Xing Mao.

**Figure 4.**
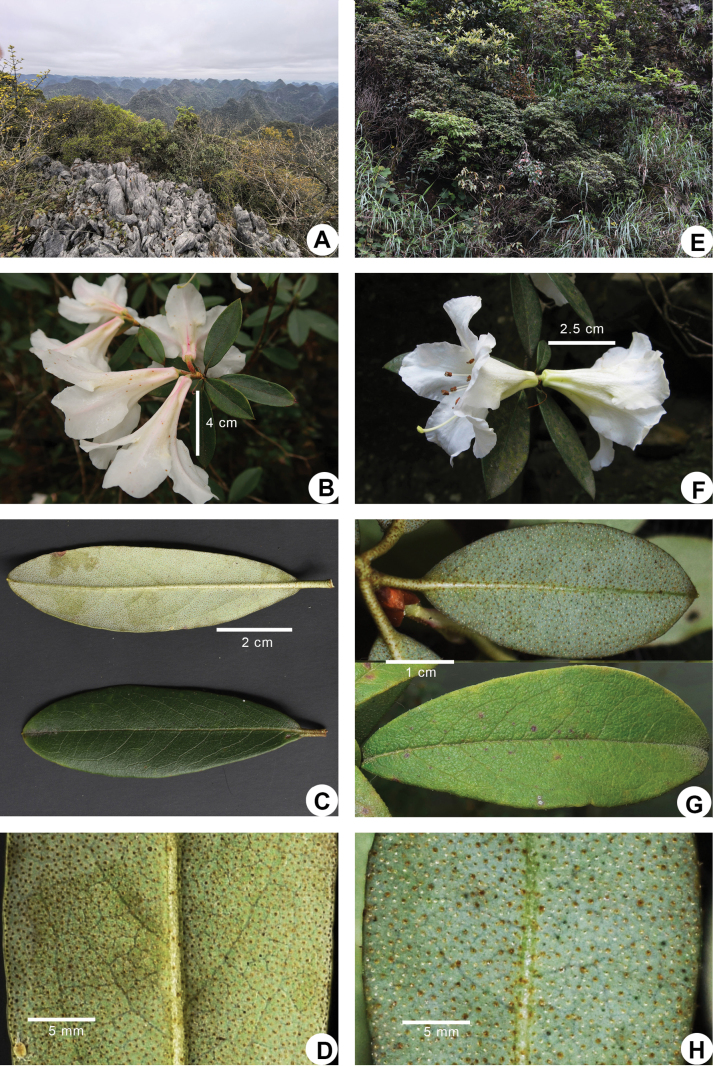
Comparisons of *Rhododendron mulunense* sp. nov. (**A–D**) and *R. wumingense* (**E–H**). **A, E.** Habitat; **B, F.** Flowers per inflorescence; **C, G.** Abaxial and adaxial leaf surface; **D, H.** Micrograph of the scales on the abaxial leaf surface. Photographs by Yu-Song Huang and Xing-Xing Mao.

Figs 1, 2, 3, 4 (A–C)

#### Type.

China. • Guangxi: Hechi City, Huanjiang County, Mulun National Nature Reserve, 25°06'N, 107°57'E, on the limestone under sparse forest, in summits of karst mountains, elev. 641 m, 3 March 2021, *Ying Qin et al., GXQY20210303021* (***holotype***: IBK! 00470814, ***isotype***: IBK! 00470813) (Fig. 1)

#### Diagnosis.

Resembles *Rhododendron wumingense* Fang but distinguishable by the absence of indumentum on young shoots, leaf margins, and petioles (vs. sparsely setose), longer and larger leaves (4.2–7.4 × 2.3–3.8 vs. 3.5–4.5 × 1.8–2.4 cm), relatively sparser scales on abaxial leaf surfaces (1–4 vs. 1–3 × their own diameter apart), corollas that are tubular funneled (vs. broadly funneled), longer and wider (5.9–7.5 × 4.7–7 vs. 3.1–4.5 × 3.3–4.9 cm) with slender tubes (2.9–3.9 × 0.5–1.2 vs. 1.9–2.5 × 1.7–2.4 cm), outer surface of corolla tubes white flushed with pink (vs. yellowish green) and pubescent but not scaly (vs. sparsely scaly and hairless) (Figs 1, 2, 4; Table 1).

**Table 1.** Comparisons of morphological characteristics, phenology, and geographic distributions of *Rhododendron mulunense* and similar species.

Characters	*R. mulunense*	*R. wumingense*	*R. pachypodum*	R. ciliicalyx subsp. lyi	*R. liliiflorum*	*R. levinei*	*R. kiangsiense*
Young shoot	hairless	sparsely setose	hairless	densely setose	hairless	sparsely setose	hairless
Leaf shape	elliptic, oblong-lanceolate	elliptic, oblong-obovate	oblong-lanceolate, obovate	oblong-lanceolate, narrowly obovate	oblong	elliptic, elliptic-obovate	oblong-elliptic
Leaf size (cm)	4.2–7.4 × 2.3–3.8	3.5–4.5 × 1.8–2.4	5.8–10.9 × 2.4–5.3	4.5–7.0 ×1.6–4.5	7.2–16.3 × 2.1–5.0	4.7–8.2 × 1.9–4.1	4.0–5.1 × 1.8–2.5
Leaf margin	hairless	densely setose	hairless	densely setose	hairless	densely setose	hairless
Scales × (own diameter apart)	1–4	1–3	1 or contiguous	0.5–1	1–3	1–1.5	1–2
Petiole	hairless	densely setose	nearly hairless	densely setose	hairless	sparsely setose	sparsely setose
Inflorescence	2–4-flowered	1–2-flowered	2–4-flowered	2–3-flowered	2–3-flowered	2–4-flowered	2-flowered
Calyx margin	hairless	hairless	sparsely setose	densely setose	hairless	densely setose	hairless
Calyx lobe length (mm)	0.5–1.0	0.7–1.2	1.0–1.6	1.0–3.0 (6.0)	10–15	8–10	7–8
Corolla shape	tubular-funnelform	broadly funnelform	broadly funnelform	broadly funnelform, funneled-campanulate	tubular-campanulate	broadly funnelform	broadly funnelform
Corolla size (cm)	5.9–7.5 × 4.7–7.0	3.1–4.5 × 3.3–4.9	4.5–6.5 × 4.2–5.8	3.7–5.7 × 3.4–5.4	8.0–9.5 × 6.5–8.3	4.5–6.1 × 4.9–5.5	4.1–6.2 × 4.3–5.1
Tube size (cm)	2.9–3.9 × 0.5–1.2	1.9–2.5 × 1.7–2.4	0.6–1.3 × 1.7–2.9	0.5–1.2 × 1.7–2.6	3.5–4.0 × 2.0–3.5	0.5–0.8 × 1.8–2.3	0.4–0.7 × 1.9–2.2
Color of corolla tube outside	white flushed with pink	yellowish green	white, sometimes flushed with pink	white flushed with pale red	white to yellowish green	white, sometimes flushed with pink	white flushed with light pink
Corolla tube outside	not scaly, pubescent	sparsely scaly, hairless	densely scaly and pubescent at base	scaly and pubescent at base	densely scaly, hairless	sparsely scaly, hairless	sparsely scaly, hairless
Flowering time	March to April	March to April	April to May	April to May	May to June	April to May	April to May
Geographic distribution	NW and SW Guangxi, SE Guizhou	Central Guangxi	SW and W Guizhou, NW, W, S, SE, and Central Yunnan	W Guizhou, NE Yunnan	N, NE, and NW Guangxi, Guizhou, Hunan, SE Yunnan	Guangxi, Guizhou, Guangdong, Fujian, Jiangxi, Hunan	Guangxi, Jiangxi, Zhejiang, Fujian

#### Description.

***Shrubs***, 0.5–2 m tall; young shoots stout, short, and relatively sparse, brownish scaly, hairless. ***Stems*** robust, erect, much-branched and sparsely scaly. ***Leaf-bud scales*** acuminate, elongated at flowering time. ***Petioles*** densely scaly, hairless, 0.5–0.8 cm long. ***Leaf*** leathery, elliptic, oblong-lanceolate, 4.2–7.4 × 2.3–3.8 cm, apex acute or obtuse, mucronate, base tapering or narrowly cuneate, margin slightly revolute and glabrous; adaxial surface green with minute and transparent scales, somewhat shining, hairless; abaxial surface usually gray-white, densely scaly, scales one to four times their own diameter apart, pale brown or brownish-red; midrib sunk adaxially, conspicuously raised abaxially, and lateral veins sunk adaxially but not obvious abaxially. ***Inflorescences*** terminal, umbellate, 3-flowered, sometimes 2- or 4-flowered; pedicel 0.5–1 cm long, densely scaly, hairless. ***Calyx*** 5-lobed, lobes 0.5–1 × 0.5–0.8 mm, rounded or triangular, outside densely to moderately scaly, margins glabrous. ***Corolla*** tubular-funnelform, 5.9–7.5 × 4.7–7 cm, narrowly tubular near the middle and lower part of corolla, 5-lobed; corolla lobes white, with yellow markings inside; ***corolla tubes*** slender, 2.9–3.9 × 0.5–1.2 cm, white flushed with pink and not scaly but pubescent outside, glabrous with yellow markings inside. ***Stamens*** 10, unequal, 3.7–5.6 mm long; filaments densely pubescent at the base. ***Ovary*** ovoid, 5-locular, 0.7–1.2 mm long, densely scaly, hairless. ***Style*s** scaly and pubescent near the base, curved upwards, extend beyond the corollas. ***Capsule*** ovoid, 1.4–2.1 × 0.4–0.7 cm, densely scaly, hairless, calyx persistent.

#### Phenology.

Flowering from March to April. Fruiting from July to August.

#### Etymology.

The specific epithet of this newly described species refers to its type localities at Guangxi Mulun National Nature Reserve, part of the South China Karst landscape registered in UNESCO’s World Heritage List.

#### Vernacular name.

Simplified Chinese: 木论杜鹃; Chinese pinyin: mù lùn dù juān.

#### Distribution and ecology.

This new species is known from five localities to date. Three localities are fragmentally distributed in the same province, including two localities in Huanjiang County, Hechi, NW Guangxi, and one smaller locality at Molu Mountain (105°52'E, 23°23'N) in Napo County, Baise, SW Guangxi. The remaining two localities were distributed at Dongli (107°59'E, 25°22'N) in Mao-lan National Nature Reserve, Libo County, S Guizhou (Fig. 3). The plants were found on limestone under sparse, mixed, broad-leaf forest on the karst mountaintop (Figs 2A, 4A) at elevations from 640 to 1350 m (Fig. 3). The associated plants include *Calocedrus rupestris* Aver., T.H.Nguyên & L.K.Phan (Cupressaceae), *Zanthoxylum dimorphophyllum* Hemsl. (Rutaceae), *Platycarya strobilacea* Siebold & Zucc. (Juglandaceae), *Polygala wattersii* Hance (Polygalaceae), *Acer sycopseoides* Chun (Sapindaceae), *Alyxia schlechteri* H.Lév. (Apocynaceae), *Leptopus pachyphyllus* X.X.Chen (Phyllanthaceae), *Rhododendron liboense* Zheng R.Chen & K.M.Lan (Ericaceae), *Paphiopedilum micranthum* Tang & F.T.Wang (Orchidaceae), and *Ophiorrhiza japonica* Blume (Rubiaceae).

#### Systematic position in *Rhododendron*.

Subsect. Maddenia Sleumer, Sect. Rhododendron, Subgen. Rhododendron.

#### Conservation status.

According to multiple fieldwork expeditions for *Rhododendron* plants around the karst mountains of Guangxi and Guizhou, five subpopulations or localities of *R. mulunense* have been discovered. Fewer than 30 mature individuals were found at two localities of less than 100×100 m^2^ in Huanjiang County, nine mature individuals were found at Molu mountain, and approximately 150 mature individuals were found in two localities at Mao-lan National Nature Reserve. Based on our investigations, the habitat quality and population size of *R. mulunense* have a continuing downward trend. Although two subpopulations, or all individuals at type localities from Huanjiang, are protected well, there are severe threats in other localities. Two subpopulations are situated in very small rocky hills in Mao-lan, with limited area encircled by urban roads, residential communities, and farmlands. Another subpopulation faces more severe threats in Napo, including its few mature individuals, limited distribution, and degraded habitat due to deforestation and wind power projects. Based on these findings, the conservation status of this new species should be assessed as Endangered (EN) under the IUCN Red List criteria C2a(i); D (Gibbs et al. 2011; IUCN 2012). This recommendation reflects the extremely limited distribution and very small population sizes of *R. mulunense*, combined with an observed continuing decline in numbers of mature individuals resulting from its close association with human activities and vulnerability to habitat loss.

Conservation studies of *Rhododendron* species indicated that the member botanic gardens of BGCI (Botanic Garden Conservation International, Richmond, UK) and the Global Conservation Consortium for *Rhododendron* play important roles in the conservation of species of subsect. Maddenia (BGCI 2020; Hu et al. 2024). Notably, a study focusing on conservation of *Maddenia* species in global botanic gardens suggested that ex situ living collections are urgently required to conserve wild endemic *Maddenia* species in countries of origin, and broader sampling of wild populations should be conducted for these threatened species (Hu et al. 2024). We propose extending the ecogeographical range of wild collections of *R. mulunense* and establishing ex situ collections in three member gardens of BGCI in China (Kunming Botanical Garden, Guilin Botanical Garden, and Lushan Botanical Garden). Meanwhile, given the threats to this endangered species, we urge local authorities to carry out habitat restoration and protection of the subpopulations in Mao-lan and Napo.

#### Additional specimens examined.

China. Guangxi Zhuang Autonomous Region: Hechi City, Huanjiang County, Chuanshan Town, Mulun National Nature Reserve, alt. 641 m, 3 March 2021, *Y. Qin et al. GXQY20210303022* (IBK), *GXQY20210303023* (IBK), *GXQY20210303024* (IBK, IBSC), *GXQY20210303025* (IBK, IBSC), *GXQY20210303026* (IBK, PE), *GXQY20210303027* (IBK, PE), *GXQY20210303028* (IBK, PE), *GXQY20210303029* (IBK); the same town, Jianfulun, alt. 1000 m, 22 April 2025, *J.H. Liang, M.L. Mo & Q.M. Wei YB0225* (IBK); the same town, Zhonglun, alt. 970 m, 29 March 2025, *Y.S. Huang Y25032906* (IBK); the same locality, elev. 980 m, 23 April 2025, *J.H. Liang, M.L. Mo & Q.M. Wei YB0264* (IBK).

#### Notes.

The species of Rhododendron subsect. Maddenia show high interspecific diversification in morphological traits, including whether shoots and leaves have indumentum, leaf shape and size, scale density on the abaxial leaf surface, corolla shape and size, indumentum and scales on the corolla and tube exterior, and the color of the corolla and tube exterior (Min and Fang 1990; Fang et al. 2005). Some of these divergences between species may be attributed to heterogeneous habitat conditions. In south China, certain *Maddenia* species growing in dry conditions or subhumid habitats generally have hairless shoots and leaves, such as *R. pachypodum* Balf.f. & W.W.Sm., *R. liliiflorum* Levl., and *R. kiangsiense* Fang, while the shoots and leaves of species growing in moister or humid conditions tend to be covered with setose hairs; for example, *R. wumingense* Fang, R. ciliicalyx subsp. lyi (Levl.) R.C.Fang, and *R. levinei* Merr. (Table 1). Therefore, species distributed across different conditions or habitats have distinct morphological traits in subsect. Maddenia. Meanwhile, this indicates that habitat conditions can be used to discriminate specific species in this group.

Among the morphological traits mentioned above, the presence or absence of indumentum on shoots and leaves, the shape and size of the corolla and tube, the presence or absence of indumentum and scales on the corolla and tube exterior, the presence or absence of indumentum on calyx margins, the size of the calyx lobe, and the scale density on the abaxial leaf surface are the most taxonomically important. Both the morphological characteristics and geographic distribution of *R. mulunense* are most similar to those of *R. wumingense*, from which it can nevertheless be readily distinguished (Figs 2, 4; Table 1). Moreover, *R. mulunense* grows on limestone under subhumid conditions with sparse forest cover (Figs 2A, B, 4A), while *R. wumingense* is found on granite rocks under moister conditions in dense forest on montane slopes (Fig. 4E) or on open wet cliffs.

**Figure 5.**
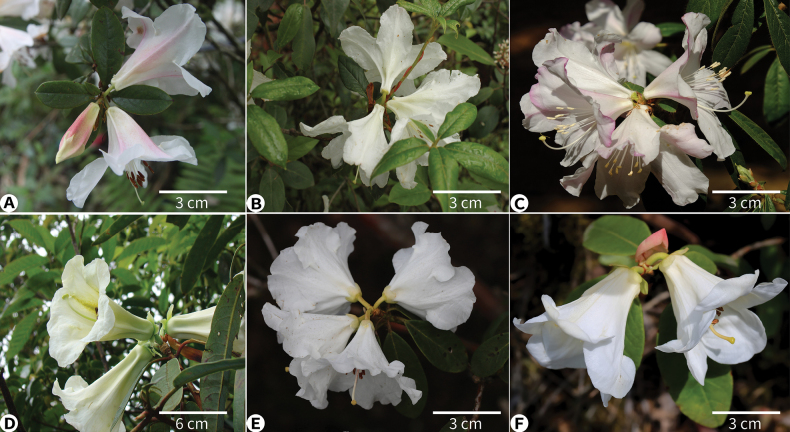
Photographs of other species similar to *Rhododendron mulunense*. **A.***R. dendricola*; **B.***R. pachypodum*; **C.**R. ciliicalyx subsp. lyi; **D.***R. liliiflorum*; **E.***R. levinei*; **F.***R. kiangsiense*. Photographs by Xing-Xing Mao, Yu-Song Huang, De-Tuan Liu, and Bing-Hua Chen.

To assess whether it should be recognized as a new species, we compared *R. mulunense* with other similar species (Fig. 5; Table 1). Morphologically, this newly discovered species is similar to *R. pachypodum* and *R. dendricola* Hutch.: it has oblong-lanceolate leaves, an undeveloped calyx, and white flowers. However, it can be distinguished from these species by its tubular-funnelform corolla with an obviously slender tube lacking scales. *R. pachypodum* has a later flowering period, and *R. dendricola* has a narrow, non-overlapping distribution in the Himalaya–Hengduan Mountains, where it generally grows among conifers (Gibbs et al. 2011). In terms of the number of flowers per inflorescence and geographical distribution, *R. mulunense* is similar to other species of subsect. Maddenia, including R. ciliicalyx subsp. lyi, *R. liliiflorum* (including the synonym *R. chunienii* Chun and W.P.Fang), *R. levinei*, and *R. kiangsiense* (Li 2008; Geng 2004, 2014; Yang et al. 2022), but it can be readily distinguished by its tubular-funnelform corolla with the tube pubescent and not scaly, its undeveloped calyx, and an earlier flowering period.

Hu et al. (2023) showed that there have been polyploidization events in multiple species within subgen. Rhododendron, which could result in phenotypes distinct from their diploid progenitors. Nevertheless, polyploidization seldom occurs in subsect. Maddenia, and all related species, including *R. wumingense*, *R. pachypodum*, *R. dendricola*, *R. ciliicalyx*, *R. liliiflorum*, *R. levinei*, and *R. kiangsiense*, have been confirmed as diploids (Hu et al. 2023). Based on these findings, our study concludes that *R. mulunense* represents a new species in subsect. Maddenia of subgen. Rhododendron. Detailed comparisons of morphology, phenology, and distributions of *R. mulunense* and related species are summarized in Table 1. In addition, an identification key is provided to facilitate discrimination of *R. mulunense* from similar *Maddenia* species.

### ﻿Key to *Rhododendron mulunense* and similar *Maddenia* species

**Table:** 

1	calyx lobes usually less than 3 mm in length (rarely much longer)	**2**
–	calyx lobes far more than 6 mm in length	**3**
2	corolla tubular-funnelform with a slender tube	***R. mulunense***
–	corolla broadly funnelform with a robust tube	**4**
3	young shoot and leaf margin sparsely to densely setose	***R. levinei***
–	young shoot and leaf margin hairless	**5**
4	scales on abaxial leaf surface generally 2–3 or 4 × their own diameter apart	**6**
–	scales on abaxial leaf surface less than one (rarely one) × their own diameter apart	**7**
5	corolla tubular-campanulate, generally 8–10 cm in length	***R. liliiflorum***
–	corolla broadly funnelform, less than 7 cm in length	***R. kiangsiense***
6	young shoot, petiole, and leaf margin sparsely or densely setose	***R. wumingense***
–	young shoot, petiole, and leaf margin hairless; usually epiphytic	***R. dendricola***
7	young shoot and leaf margin hairless, calyx margin sparsely setose	***R. pachypodum***
–	young shoot and leaf margin densely setose, calyx margin setose	**R. ciliicalyx subsp. lyi**

## Supplementary Material

XML Treatment for Rhododendron mulunense

## ﻿Additional information

### Use of AI

No use of AI was reported.

### Author ORCIDs

Ying Hou https://orcid.org/0000-0002-6111-3485

Yu-Song Huang https://orcid.org/0000-0003-2693-8518

Liu-Juan Luo https://orcid.org/0009-0000-9115-1346

Xing-Xing Mao https://orcid.org/0009-0006-2313-8004

### Data availability

All of the data that support the findings of this study are available in the main text.
